# Defining the Optimal Radiation-induced Lymphopenia Metric to Discern Its Survival Impact in Esophageal Cancer

**DOI:** 10.1016/j.ijrobp.2024.12.014

**Published:** 2025-01-02

**Authors:** Pim J.J. Damen, Max Peters, Brian Hobbs, Yiqing Chen, Uwe Titt, Remi Nout, Radhe Mohan, Steven H. Lin, Peter S.N. van Rossum

**Affiliations:** a Department of Radiation Oncology, The University of Texas MD Anderson Cancer Center, Houston, Texas;; b Department of Radiation Oncology, Amsterdam University Medical Center, Amsterdam, The Netherlands;; c Department of Radiotherapy, Erasmus Medical Center Cancer Institute, University Medical Center Rotterdam, Rotterdam, The Netherlands;; d Department of Radiotherapy, Radiotherapiegroep, Deventer, The Netherlands;; e Department of Population Health, Dell Medical School, The University of Texas at Austin, Austin, Texas;; f Department of Biostatistics and Data Science, University of Texas Health Science Center, Houston, Texas;; g Department of Radiation Physics, The University of Texas MD Anderson Cancer Center, Houston, Texas

## Abstract

**Purpose:**

A detrimental association between radiation-induced lymphopenia (RIL) and oncologic outcomes in patients with esophageal cancer has been established. However, an optimal metric for RIL remains undefined but is important for the application of this knowledge in clinical decision-making and trial designs. The aim of this study was to find the optimal RIL metric discerning survival.

**Methods and Materials:**

Patients with esophageal cancer treated with concurrent chemoradiation therapy (CRT; 2004–2022) were selected. Studied metrics included absolute lymphocyte counts (ALCs) and neutrophil counts—and calculated derivatives—at baseline and during CRT. Multivariable Cox regression models for progression-free survival (PFS) and overall survival (OS) were developed for each RIL metric. The optimal RIL metric was defined as the one in the model with the highest c-statistic.

**Results:**

Among 1339 included patients, 68% received photon-based and 32% proton-based CRT (median follow-up, 24.9 months). In multivariable analysis, the best-performing models included “ALC in week 3 of CRT” (corrected c-statistic 0.683 for PFS and 0.662 for OS). At an optimal threshold of <0.5 × 10^3^/*μ*L (ie, grade ≥3 RIL), ALC in week 3 was significantly associated with PFS (adjusted hazard ratio, 1.64; 95% CI, 1.27–2.13) and OS (adjusted hazard ratio, 1.56; 95% CI, 1.15–2.08), with 5-year PFS of 29% vs 40% and OS of 38% vs 51%, respectively.

**Conclusions:**

Reaching grade ≥3 RIL in week 3 of CRT for esophageal cancer is the strongest RIL metric to distinguish survival outcomes. We suggest that this metric should be the target for lymphopenia-mitigating strategies and propose this metric to be included in future trials.

## Introduction

Esophageal cancer is the sixth leading cause of cancer-related mortality worldwide, with more than 500,000 estimated deaths.^1^ The standard of treatment for locally advanced esophageal cancer consists of chemoradiation therapy (CRT), followed by surgery in operable patients.^2^ Recently, adjuvant immunotherapy has been shown to prolong disease-free survival in patients with an incomplete resection after CRT and surgery.^3^ An alternative standard of treatment in esophageal adenocarcinoma is perioperative triple chemotherapy without radiation therapy (RT).^4^

Lymphocytes are a crucial part of the human immune system and are pivotal in the antitumor immune response and surveillance.^5,6^ However, preclinical and clinical studies have shown that even low doses of RT (<1 Gy) directly kill circulating lymphocytes, making them the most radiosensitive cells of the hematopoietic system.^7–9^ In recent years, it has been hypothesized that the antitumor effect of radiation is counteracted by this radiation-induced lymphopenia (RIL). In esophageal cancer, studies have established the detrimental prognostic association between RIL and pathologic response,^10–14^ progression-free survival (PFS)^10,13,15–19^ and overall survival (OS).^10,15,17–21^ RIL is, therefore, an area of growing interest, especially because immunotherapy has become such an integral part of cancer therapy, which is largely reliant on intact lymphocytes as a part of the adaptive immunity system.^22^

To apply this knowledge to clinical decision-making and for the design of lymphopenia-mitigating trials, it is of considerable importance to determine an optimal metric for RIL. Such a metric for RIL remains undefined in current literature but, in principle, would be easy to measure, easy to compare across studies, and most impactful in terms of discerning survival outcomes independently of other prognostic factors. For thoracic tumors (ie, lung and esophageal cancer), most studies use a threshold for the lowest absolute lymphocyte count (ALC), either during treatment (ALCnadir),^12–15,17,19–21,23–26^ or the first post-RT measurement.^27,28^ Most studies use predefined thresholds according to the National Cancer Institute Common Terminology Criteria for Adverse Events (CTCAE),^29^ including grade ≥3 RIL (ie, <0.5 ×10^3^/*μ*L; most commonly reported metric in lung cancer)^23–25^ or grade 4 lymphopenia (ie, <0.2 × 10^3^/*μ*L; most commonly reported metric in esophageal cancer).^12,13,16,17,19–21^ Others have selected data-driven thresholds for RIL that comprise the optimal value to maximize survival curve differences.^14,15,26,27^ In addition, some use the neutrophil-to-lymphocyte ratio (NLR) rather than ALC values.^11,30–32^ Finally, another metric of lymphopenia that might correlate well to survival is the speed of lymphocyte depletion, which can be quantified by absolute or relative differences between ALC counts or NLR during treatment as compared to those values at baseline (ie, ΔALC and ΔNLR).^11,31,32^

The use of various RIL metrics in literature can be partly explained by relatively small study sample sizes, preventing proper comparison of multiple RIL metrics within one study due to multiple testing issues. Therefore, the aim of this largest study of its kind in patients who underwent CRT for esophageal cancer was to find the optimal lymphopenia metric that—independent of other prognostic factors—best distinguishes patients in terms of PFS and OS.

## Material and Methods

This retrospective cohort study was approved by the institutional review board of our institution. The requirement to obtain informed consent was waived. The study adhered to the Health Insurance Portability and Accountability Act and the principles of Good Clinical Practice.

### Study population

From a single-center retrospective database, patients with biopsy-proven esophageal cancer treated with concurrent CRT between January 2004 and September 2022 were selected. RT generally consisted of a total dose of 50.4 Gy in 28 fractions delivered by either proton beam therapy (PBT) or intensity modulated RT. Concurrent chemotherapy generally consisted of a taxane-, platinum-, or fluoropyrimidine-based doublet regimen. Patients with <90 days of follow-up, overall CRT treatment time >50 days, simultaneous irradiation of a second primary tumor, a planned total dose other than 41.4 to 50.4 Gy, concurrent immunotherapy, or a missing baseline ALC value were excluded. In addition, patients with no available ALC value in weeks 4, 5, or 6 during CRT were excluded because the lowest ALC values typically occur in these latter weeks of treatment.

### Lymphopenia metrics

Full blood count data were collected at baseline and weekly during CRT. Studied RIL metrics included ALC values and neutrophil counts—and calculated derivatives—both at baseline and during CRT (ie, baseline NLR values, ALCnadir values, grade ≥3 RIL [<0.5 × 10^3^/*μ*L], and grade 4 RIL [<0.2 × 10^3^/*μ*L] during CRT according to CTCAE criteria, ALC values and neutrophil counts per week, maximum NLR values, maximum neutrophil count, and both absolute and relative ΔALC and ΔNLR values for each week during CRT compared with baseline).

### Survival outcomes

The outcomes of this study included PFS and OS and were calculated from the start date of CRT to disease progression or death, respectively, and censored at the date of the last follow-up.

### Statistical analysis

Descriptive statistics of patient-, tumor-, and treatment-related baseline characteristics were presented. The following steps were performed in accordance with the transparent reporting of a multivariable prediction model for individual prognosis or diagnosis statements.^33^ Missing data were considered missing at random and handled with multiple imputations using chained equations. A total of 100 new sets were created. During the imputation process, highly collinear variables were excluded from the predictor matrix. The outcome was included in the imputation procedure in the form of the Nelson-Aalen estimator.

Univariable Cox proportional hazard regression models were created to study the crude association of the RIL metrics with PFS and OS. In multivariable Cox proportional hazard regression analyses, the association of each RIL metric with PFS and OS was subsequently adjusted for known prognostic factors, including age, body mass index (BMI, kg/m^2^), gender, the Eastern Cooperative Oncology Group performance status, smoking status, histology, and clinical TNM stage. To prevent the problem of multicollinearity, not >1 RIL metric was included in a model at a time, and we ascertained that the clinical variables were not highly correlated (Spearman rho <0.6).^33^

Model performances were evaluated by calculating Akaike information criterion (AIC)—a statistical measure used to compare models by balancing goodness-of-fit with model complexity—for each model. Subsequently, a selection was made of the 5 most promising models, which were defined as the 5 models with the lowest AIC values. Time-dependent c-statistics—measures used to evaluate the discriminative ability of survival models over time—were calculated for these 5 most promising models and also for 4 commonly applied models that included either baseline ALC, grade ≥3 RIL, grade 4 RIL, or ALCnadir values. These latter ALC metrics are the most commonly reported in thoracic cancer literature. The c-statistics were corrected for optimism (ie, to account for overfitting) by internal validation using 2000 bootstrap resamples in which the specific multivariable model was recreated. Among the 9 remaining models (ie, the 5 most promising models and the 4 commonly applied models), the most optimal RIL metric was identified by selecting the metric that provided the model with the highest corrected c-statistic. To study the potential impact of the previous multiple imputation steps on the final model performance, a sensitivity analysis was performed excluding all cases with one or more missing predictor values.

For each of the 9 remaining RIL metrics, an optimal threshold was determined that maximized both the PFS and OS differences between patient groups. Kaplan-Meier curves for PFS and OS were generated based on these threshold values. Finally, for the most optimal RIL metric and grade 4 RIL, an exploratory comparison between photon and proton therapy was performed.

Statistical analyses were performed using R Studio 4.3.0, open-source software (http://www.R-project.org; “survival,” “survminer,” “mice,” and “rms” packages), and SPSS (version 28.0, IBM Corp). To adjust for multiple testing, the *P* value for significance was set to 0.001.

## Results

Out of 1484 patients with esophageal cancer treated with CRT in the study period, a total of 1339 patients were eligible for inclusion. The flow of patient selection is shown in Figure E1. The baseline characteristics are presented in Table 1. Cutaneous cancers, including basal and squamous cell cancers, were counted as secondary malignancies. Among the 1339 patients, 620 (46.3%) underwent surgery after CRT. Only 10 patients (0.7%) received adjuvant immunotherapy after completion of CRT and surgery.

The median follow-up duration for the whole group was 24.9 months. The average baseline ALC value was 1.59 × 10^3^/*μ*L. The average ALC values declined by 32% in the first week of CRT and subsequently by 29% from week 1 to 2, 31% from week 2 to 3, 23% from week 3 to 4, 17% from week 4 to 5, and 5% from week 5 to 6.

Results of univariable Cox regression analyses of all baseline characteristics and RIL metrics for PFS and OS are provided in Table 2. Studied RIL metrics that were significantly related to both PFS and OS in univariable analyses were BMI (kg/m^2^), World Health Organization performance status, smoking status, histology, and clinical TNM stage.

Certain variables were excluded from the multivariable analysis due to collinearity. Ultimately, each multivariable model consisted of age, BMI, gender, World Health Organization performance status, smoking status, histology, clinical TNM stage, surgery (yes vs no), and one of the studied RIL metrics. The calculated AIC values for all multivariable models are provided in Table E1.

The 5 most promising multivariable models (with the lowest AIC scores) included those with the following RIL metrics: ALC in week 3 of CRT, ΔALC (%) in week 3, absolute neutrophil count (ANC) at baseline, NLR in week 1, and NLR in week 3 of CRT. The corrected c-statistics of these models, as well as for the models, including the 4 most commonly applied RIL metrics (ie, baseline ALC values, grade ≥3 RIL, grade 4 RIL, and ALCnadir values), are presented in Table 3. The most optimal RIL metric (ie, with the best ability to discriminate between outcomes) was the ALC values in week 3 of CRT, with the highest corrected time-dependent c-statistic of 0.683 for PFS and 0.662 for OS. The models for NLR in week 3 and ΔALC (%) in week 3 provided very similar model performance and c-statistics for PFS and OS prediction. On the other hand, results for grade ≥3 RIL (in any week), grade 4 RIL, and ALCnadir values were slightly inferior to those of ALC values in week 3 (Table 3). For prediction at baseline, the performance of multivariable models with ALC or ANC at baseline was fair, with corrected c-statistics of 0.677 and 0.679 for PFS and 0.656 and 0.660 for OS, respectively.

Sensitivity analyses excluding all cases with one or more missing predictor values for the multivariable models with ALC values in week 3 (n = 1114) resulted in closely resembling model performances for both PFS and OS (corrected c-statistic 0.684 and 0.661, respectively; Table E2). The complete case analyses for the models with grade 4 RIL (n = 1161) also resulted in comparable model performances for both PFS and OS (corrected c-statistic 0.678 and 0.654, respectively; Table E3).

**Fig. 1.**
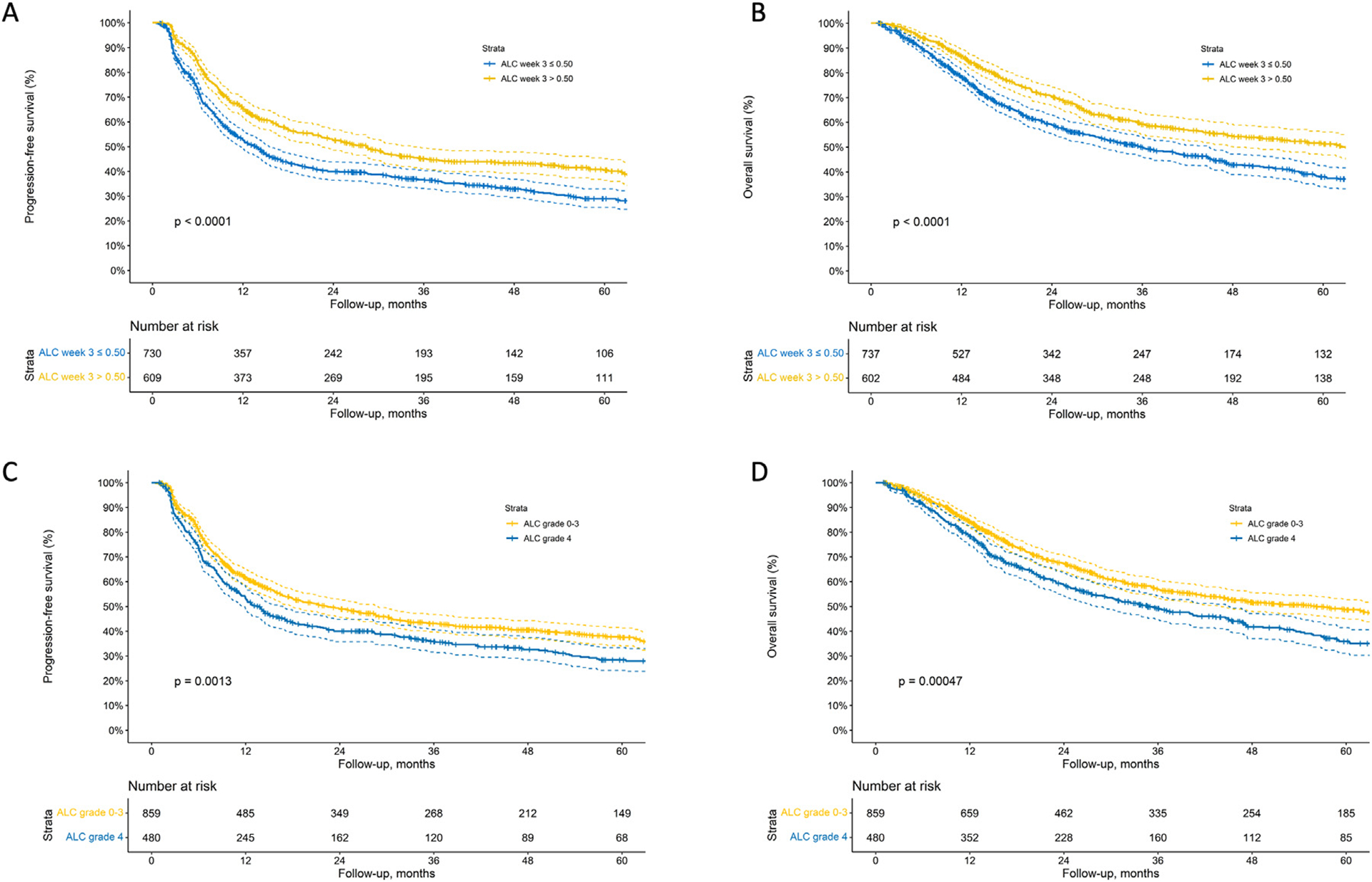
Kaplan-Meier analysis for absolute lymphocyte count in week 3 for (A) progression-free survival and (B) overall survival and for grade 4 lymphopenia for (C) progression-free survival and (D) overall survival.

The optimal threshold for ALC in week 3 that maximized the survival difference was determined at <0.5 × 10^3^/*μ*L (which conveniently corresponds to grade ≥3 RIL according to CTCAE). At this threshold of grade ≥3 RIL in week 3 of CRT, RIL was independently and significantly associated with PFS (adjusted hazard ratio [aHR], 1.64; 95% CI, 1.27–2.13) and OS (aHR, 1.56; 95% CI, 1.15–2.08, respectively). In 716 patients with an ALC value in week 3 of <0.5 × 10^3^/*μ*L (ie, with grade ≥3 RIL), 5-year PFS and OS rates were 29% and 38%, respectively, versus 40% and 51% in 621 patients with ALC values in week 3 of ≥0.5 × 10^3^/*μ*L (Fig. 1A, B). For comparative purposes, for patients with grade 4 RIL during CRT (ie, the most commonly applied RIL metric in current literature), 5-year PFS and OS rates were 28% and 36%, versus 37% and 49% for patients without grade 4 RIL, respectively (Fig. 1C, D). The Kaplan-Meier curves for PFS and OS of the other multivariable models are provided in Figure E2.

In 68% of patients, photon therapy and in 32% PBT were used as RT techniques. The only significant differences between the photon and proton therapy groups were mean age (respectively, 62.1 vs 66.9 years; *P* < .001) and surgery after CRT (respectively, 47.1% vs 40.3%; *P* = .020). Patients treated with PBT experienced significantly less grade ≥3 RIL in week 3 (37.7% vs 57.2%; *P* < .001) and grade 4 RIL (21.2% vs 42.7%; *P* < .001) compared with patients treated with photon therapy.

## Discussion

For research purposes and future clinical decision-making, consistent use of an ideal RIL outcome measure is of importance, but in current literature, no consistency nor consensus exists on the best metric to define RIL. Our study demonstrates that the ALC value in week 3 of CRT for esophageal cancer (at a threshold of 0.5 × 10^3^/*μ*L, equivalent to grade ≥3) is the strongest metric for RIL to distinguish survival outcomes, even after correction for known prognostic factors. We suggest that this metric should be reported in trials, and potentially used as the target for lymphopenia-mitigating strategies.

Although the ALC values in week 3, at a threshold of grade ≥3 lymphopenias, appeared to be the best predictor in this study, other factors (eg, baseline ALC values) came close in their performance to distinguish survival and could also be considered for RIL mitigation strategies at baseline. The baseline ALC value is also a reasonable predictor of survival and could serve as a selection criterion as well. For esophageal cancer, most studies in the literature use the CTCAE grade 4 lymphopenia metric any week during treatment.^12,13,17,19–21^ Our study showed that this metric is indeed predictive of survival with a corrected c-statistic of 0.677 for PFS and 0.657 for OS, but the metric lost statistical significance for PFS and OS once adjusted for confounders (*P* = .426 and *P* = .259, respectively). On the other hand, ALC values in week 3 of CRT (at a threshold of 0.5 × 10^3^/*μ*L) were slightly more predictive with a corrected c-statistic of 0.683 for PFS and 0.662 for OS, and did remain significant in multivariable analysis for PFS and OS (*P* < .001 and *P* = .004, respectively). An additional benefit of the “ALC value in week 3” is that this metric is already known in the third week of CRT. Although the models for NLR values in week 3 and ΔALC% in week 3 provided very similar model performance for the prediction of PFS (corrected c-statistic 0.682 and 0.682, respectively) and OS (0.662 and 0.663, respectively), ALC values in week 3 were finally selected as the best metric because of their simplicity, because no additional ANC value or calculation is needed. Also, if the desire is to distinguish at baseline, baseline ALC can be used for the prediction of PFS (corrected c-statistic 0.677) and OS (0.656).

The mechanism underlying the interesting finding that an early ALC values measurement in week 3 suffices remains unclear. It is possible that this metric is the best measure for intrinsic radiosensitivity because this measurement was generally taken on day 1 of week 3, which is only 14 days after the start of CRT. It is possible that only from that point onward the bone marrow starts to compensate adequately through repletion, making ALC values in weeks 4 and 5 more multifactorial.^34^ Therefore, our study results might apply to other malignancies, but further research is required to confirm this.

Currently, several methods to potentially mitigate the risk of RIL are under study to decrease the unintentional exposure of the circulating blood pool and secondary lymphoid organs to RT. For instance, radiation dose planning can involve active avoidance of dose in the large vessels, heart, and lungs, as well as lymphocyte-rich risk organs at risk (OARs), such as the lymph nodes, spleen, and, in children, the thymus.^18,35^ However, to date, no standardized dose constraints are available in the literature. A recent systematic review provided a synopsis of the current literature on the dosimetric predictors of RIL in solid tumors, which may serve as a starting point for future clinical trials to limit RIL risk.^36^ Because these constraints are not validated in prospective trials, the current practice should still work with the “as low as reasonably achievable” principle for the OARs.^35^

Other lymphopenia-mitigating strategies include reducing the number of radiation fractions (hypofractionation) and/or the total treatment time, reducing the field size, or reducing the integral body dose. PBT is known for its steep dose gradient with generally a lower integral body and OAR dose compared with photon-based RT.^37^ The lymphocyte-sparing effect of PBT was first described retrospectively and later confirmed in a phase 2 randomized trial in patients with esophageal cancer, in which the incidence of grade 4 lymphopenia was reduced to 27.3% with proton therapy compared with 52.5% with photon-based RT (*P* = .010).^38,39^ Our study found a similar lymphocyte-sparing effect as patients treated with PBT experienced significantly less grade ≥3 RIL in week 3 (37.7% vs 57.2%; *P* < .001) and grade 4 RIL (21.2% vs 42.7%; *P* < .001) compared with patients treated with photon therapy. In contrast to other series that showed a potential survival benefit of protons related to lymphopenia mitigation,^40,41^ our study found no survival benefit for the proton therapy group. This could be related to higher patient age and less surgery in this cohort compared with the photon therapy group. Another method to reduce radiation field size is (magnetic resonance imaging- or computed tomography-based) adaptive RT.^42,43^ By daily localization of the tumor and OARs, the target can be determined more accurately, which means that conventional margins can be significantly reduced. In a more exploratory manner so far, FLASH RT seems capable of reducing exposure of circulating lymphocytes to a lethal dose by its highly increased dose rate with the dose delivered in a very short time (<0.1 seconds).^44^ Other experimental methods to reduce RIL risk include the administration of interleukin-7^45^ or the preradiation harvesting and postradiation reinfusion of lymphocytes.^46^ Finally, one approach to prevent RIL in patients at high risk of RIL might be to opt for chemotherapy, which has a greater impact on neutropenia rather than lymphopenia. Recent trials suggest that chemotherapy may be as^47^ or even more^4^ effective than neoadjuvant CRT in patients with esophageal adenocarcinoma.

Our finding of this apparently most meaningful RIL metric of “ALC in week 3” could have different clinical implications. The above-mentioned lymphopenia-mitigating strategies, if not from baseline in all patients, might be applied in selected patients only as soon as week 3 of CRT, with no need to wait until the end of treatment to finally assess the ALCnadir value in an individual patient and the occurrence or absence of grade 4 lymphopenia. Moreover, even as early as week 2 could be appropriate for patient selection for mitigation because the ALC value in week 2 was also highly predictive. Another implication of this finding could involve this metric as an endpoint in trials. For example, in most trials with RIL as an endpoint so far, grade 4 lymphopenia was chosen as an outcome,^37^ but grade ≥3 lymphopenia in week 3 might be more meaningful in terms of survival impact, and reporting of this metric is therefore recommended.

Several recent studies suggest a relationship between severe RIL and worse oncological outcomes. For example, in pooled data from 819 patients from 3 studies, a significantly lower chance of a pathologic complete response after RT was observed in patients with esophageal cancer who developed severe RIL (grade 4) during neoadjuvant CRT compared with patients without grade 4 RIL (odds ratio [OR], 0.44; 95% CI, 0.30–0.66).^10^ That meta-analysis also pooled data from 1660 patients with esophageal cancer from 4 studies and showed that grade 4 RIL was associated with a significantly worse PFS (hazard ratio [HR], 1.70; 95% CI, 1.39–2.07).^10^ Furthermore, in a different meta-analysis, pooled data were analyzed from 2733 patients with head and neck cancer from 8 studies and showed that grade ≥3 RIL was a significant risk factor for disease progression (HR, 3.16; 95% CI, 1.77–5.63).^48^

Besides treatment response and disease progression, RIL also appears to affect OS. A recent meta-analysis of 21 cohorts in 20 studies on the association between RIL and OS in patients with solid tumors showed a significantly increased risk of death for patients with grade ≥3 compared with grade 0 to 2 lymphopenia (pooled HR, 1.65; 95% CI, 1.43–1.90), as well as for patients with grade 4 compared with grade 0 to 3 lymphopenia (pooled HR, 1.50; 95% CI, 1.24–1.90).^18^ That meta-analysis only included studies that, in a multivariable manner, corrected their HRs for other prognostic factors such as age and tumor stage. Subgroup analyses confirmed the significant association between RIL and detrimental OS, separately for brain tumors, lung cancer, esophageal cancer, and pancreatic cancer.^18^ Other tumor-specific meta-analyses also showed a significant relationship between RIL and reduced OS. The aforementioned meta-analyses by Dai et al^48^ demonstrated this relationship with OS for grade 4 RIL in esophageal cancer (pooled HR, 1.50; 95% CI, 1.29–1.75)^10^ and for grade ≥3 in head and neck tumors (pooled HR, 2.94; 95% CI, 1.83–4.74). Furthermore, Upadhyay et al^49^ showed a similar relationship of OS in pooled data from 10 lung cancer studies with severe lymphopenia (pooled HR, 1.59; 95% CI, 1.40–1.81).

A relevant finding since the advent of immunotherapy is that a reduction in the number of lymphocytes also appears to result in a reduction in the effectiveness of lymphocyte-activating immunotherapeutic agents.^50–53^ A study on 167 patients with esophageal squamous cell carcinoma who received immune checkpoint inhibitors therapy showed that the occurrence of treatment-related lymphopenia before or during immune checkpoint inhibitors treatment was significantly increased by prior RT (OR, 0.50; 95% CI, 0.26–0.95) and was associated with shorter PFS (HR, 1.86; 95% CI, 1.17–2.93).^52^ Another study in 105 patients with recurrent metastatic esophageal cancer treated with immunotherapy showed that lymphopenia (ALC value, ≤0.63 ×10^3^/*μ*L) within 1 week before immunotherapy was associated with poorer OS (aHR, 1.77; 95% CI, 1.05–2.99).^53^ Grade 4 lymphopenia during previous RT was highly associated with developing preimmunotherapy lymphopenia (OR, 10.81; 95% CI, 2.19–53.47). In other thoracic tumors, a study on 309 patients with non-small cell lung cancer (NSCLC) treated with concurrent CRT with or without adjuvant durvalumab showed that durvalumab only significantly prolonged PFS in patients without severe RIL (<0.23 × 10^3^/*μ*L), and not in patients with severe RIL.^15^ In another study, investigators observed in 110 patients with NSCLC, melanoma, or renal cell carcinoma treated with RT prior to immunotherapy that grade ≥3 RIL at the initiation of immunotherapy was significantly associated with increased mortality (HR, 2.1; 95% CI, 1.1–3.9).^50^ Furthermore, Friedes et al^51^ showed in 78 patients with NSCLC after CRT that patients with versus without grade ≥3 RIL had significantly worse PFS (HR, 4.90; 95% CI, 1.70–14.2).

In addition to the above disadvantages of RT prior to immunotherapy, there are also potential advantages of combining RT and immunotherapy. Cancer damage induced by RT promotes the recognition of tumor cells by dendritic cells and triggers a cytotoxic T-cell response.^54^ This might actually increase the local and abscopal activity of immunotherapy. The pooled analysis of 2 randomized trials (PEMBRO-RT and MDACC) showed that the addition of RT to pembrolizumab in metastatic NSCLC patients significantly enhanced response rates of unirradiated lesions (OR, 2.96; 95% CI, 1.42–6.20).^55^ Consequently, this led to significantly prolonged PFS (HR, 0.67; 95% CI, 0.45–0.99) and OS (HR, 0.67; 95% CI, 0.54–0.84). Finding the optimal balance between minimizing immunosuppressive effects and promoting the immunostimulating effects of RT is an important new goal to improve treatment outcomes.

A few limitations apply to this study. Although we report on one of the largest esophageal datasets in the literature, a limitation of this study is its retrospective and single-center nature. External validation of the survival impact of the proposed RIL metric is desired and welcomed. In addition, no inferences on potential causality between RIL metrics and survival can be made based on the current study because this was not a prospective interventional study.

In conclusion, the ALC in week 3 of CRT for esophageal cancer (at a threshold of 0.5 × 10^3^/*μ*L) is the strongest metric for radiation-induced lymphopenia to distinguish survival outcomes, even after adjustment for confounders. We suggest that this easy-to-use metric should be reported in the evaluation of lymphopenia-mitigating strategies in esophageal cancer RT and encourage its use in future trials.

## Supplementary Material

Suppl

Supplementary material associated with this article can be found in the online version at doi:10.1016/j.ijrobp.2024.12.014.

## Figures and Tables

**Table 1:** Baseline characteristics

Characteristic	Patients (n = 1339)
Male gender	1117 (83.4%)
Age (y)	63.5 ± 10.7
Performance status	
WHO 0	445 (33.2%)
WHO 1	807 (60.3%)
WHO 2	85 (6.3%)
WHO 3	2 (0.1%)
BMI mean	26.5 ± 5.8
Hypertension	176 (13.1%)
Cardiac comorbidity	26 (1.9%)
Pulmonary comorbidity	14 (1.0%)
Reflux	95 (7.1%)
Diabetes	23 (1.7%)
Second malignancy	347 (25.9%)
Missing	2
Smoking	
Never	349 (26.1%)
Past/quit	648 (48.4%)
Active smoker	188 (14.0%)
Missing	154
Tumor location	
Cervical / upper	82 (6.1%)
Middle	148 (11.1%)
Lower	1106 (82.6%)
Missing	3
Histology	
Adeno	1074 (80.2%)
SCC	255 (19.0%)
Other	9 (0.7%)
Missing	1
T stage	
1	31 (2.3%)
2	143 (10.7%)
3	1083 (80.9%)
4	51 (3.8%)
Missing	31
N stage	
0	428 (32.0%)
1	513 (38.3%)
2	304 (22.7%)
3	57 (4.3%)
Missing	37
M stage	
0	1246 (93.1%)
1	78 (5.8%)
Missing	15
Clinical stage (AJCC 7^th^ edition)	
I	61 (4.6%)
II	406 (30.3%)
IIIA	432 (32.3%)
IIIB	249 (18.6%)
IIIC	80 (6.0%)
IV	79 (5.9%)
Missing	32
Modality	
3DCRT	12 (0.9%)
IMRT	631 (47.1%)
VMAT	268 (20.0%)
PSPT	382 (28.6%)
IMPT	38 (2.8%)
Missing	3
Induction chemotherapy	435 (32.5%)
Prescribed total dose (Gy)	50.4 (50.4–50.4)
Prescribed dose/fraction (Gy)	1.8 (1.8–1.8)
Prescribed number of fractions	28 (28–28)
Baseline ALC	1.50 (1.17–1.93)
Baseline ANC	4.20 (3.22–5.53)
PTV volume	593 (429–797)
Missing	273
Surgery	620 (46.3%)
Missing	3

*Abbreviations:* AJCC = American Joint Committee on Cancer; ALC = absolute lymphocyte count; ANC = absolute neutrophil count; BMI = body mass index; IMPT = intensity modulated proton therapy; IMRT = intensity modulated radiation therapy; PSPT = passive scattering proton therapy; SCC = squamous cell carcinoma; 3DCRT = 3-dimensional conformal radiation therapy; VMAT = volumetric modulated arc therapy; WHO = World Health Organization.

**Table 2:** Univariable and multivariable Cox proportional hazard regression analysis for PFS and OS

Characteristic	Univariable				Multivariable			
	PFS HR (95% CI)	*P* value	OS HR (95% CI)	*P* value	PFS aHR (95% CI)	*P* value	OS aHR (95% CI)	*P* value
Age (y)	1.01 (1.00–1.01)	.131	1.01 (1.00–1.02)	.008	0.99 (0.99–1.00)	.092	1.00 (0.99–1.01)	.627
BMI (kg/m^2^)	0.98 (0.97–0.99)	<.001*	0.96 (0.95–0.98)	<.001*	0.97 (0.96–0.98)	<.001*	0.96 (0.95–0.98)	<.001*
Gender								
Female	Reference		Reference		Reference		Reference	
Male	1.23 (1.02–1.49)	.034	1.17 (0.94–1.44)	.154	1.59 (1.24–1.91)	<.001*	1.44 (1.13–1.84)	.003
Performance status								
WHO 0	Reference		Reference		Reference		Reference	
WHO 1	1.11 (0.96–1.29)	.161	1.23 (1.04–1.45)	0.018	0.94 (0.80–1.10)	.442	1.05 (0.88–1.25)	.616
WHO 2	1.51 (1.16–1.99)	.003	1.78 (1.32–2.38)	<.001*	1.00 (0.75–1.33)	.998	1.20 (0.88–1.64)	.256
Hypertension	1.19 (0.99–1.44)	.065	1.15 (0.93–1.41)	.198				
Cardiac comorbidity	0.90 (0.55–1.48)	.679	0.89 (0.52–1.55)	.688				
Pulmonary comorbidity	1.22 (0.63–2.35)	.557	1.39 (0.69–2.80)	.352				
Diabetes	1.76 (1.13–2.74)	.013	1.27 (0.76–2.12)	.364				
Reflux	0.89 (0.68–1.16)	0.374	0.89 (0.66–1.19)	.433				
Secondary malignancy	1.01 (0.86–1.17)	0.925	1.07 (0.90–1.27)	.438				
Smoking status								
No smoking	Reference		Reference		Reference		Reference	
Past smoker	1.05 (0.89–1.24)	0.532	1.16 (0.96–1.40)	.120	1.01 (0.85–1.20)	.879	1.07 (0.88–1.30)	.513
Active smoker	1.23 (0.99–1.53)	0.058	1.31 (1.03–1.67)	.029	1.07 (0.91–1.43)	.258	1.16 (0.90–1.49)	.248
Tumor location								
Cervical	Reference							
Mid	1.22 (0.88–1.70)	0.238	1.01 (0.71–1.45)	.944				
Low	0.90 (0.68–1.19)	0.449	0.78 (0.58–1.05)	.106				
Histology								
Adenocarcinoma	Reference		Reference		Reference		Reference	
SCC	1.24 (1.05–1.47)	.014	1.29 (1.06–1.56)	.009	0.87 (0.70–1.05)	.134	0.89 (0.71–1.11)	.295
Other	3.11 (1.55–6.26)	.001	3.23 (1.60–6.51)	.001	3.54 (1.79–7.38)	<.001*	3.40 (1.67–6.94)	.001
Clinical T stage								
T1–2	Reference		Reference		Reference		Reference	
T3	1.79 (1.43–2.26)	<.001*	1.98 (1.52–2.59)	<.001*	1.54 (1.25–2.02)	<.001*	1.76 (1.33–2.32)	<.001*
T4	2.69 (1.80–4.01)	<.001*	3.32 (2.14–5.15)	<.001*	1.95 (1.20–2.81)	<.001*	2.31 (1.46–3.67)	<.001*
Clinical N stage								
N0	Reference							
N1	1.46 (1.24–1.73)	<.001*	1.54 (1.27–1.87)	<.001*	1.32 (1.11–1.57)	0.002	1.41 (1.15-	.001
N2	1.77 (1.47–2.13)	<.001*	1.82 (1.48–2.24)	<.001*	1.46 (1.21–1.78)	<.001*	1.71)	<.001*
N3	2.01 (1.43–2.82)	<.001*	1.77 (1.18–2.63)	.005	1.49 (1.08–2.19)	.017	1.47 (1.18–1.83)	.054
Clinical M stage								
M0	Reference		Reference		Reference		Reference	
M1	2.31 (1.80–2.97)	<.001*	1.88 (1.42–2.50)	<.001*	1.39 (1.01–1.72)	.039	1.18 (0.87–1.59)	.283
Clinical stage (AJCC 7th)								
IA-B	Reference		Reference					
IIA-B	1.55 (1.03–2.33)	.034	1.58 (1.00–2.52)	0.052				
IIIA	2.22 (1.49–3.32)	<.001*	2.46 (1.56–3.89)	<.001*				
IIIB	2.47 (1.64–3.73)	<.001*	2.72 (1.70–4.35)	<.001*				
IIIC	2.55 (1.59–4.10)	<.001*	3.00 (1.76–5.11)	<.001*				
IV	4.53 (2.88–7.12)	<.001*	3.93 (2.34–6.58)	<.001*				
Total dose	1.00 (0.98–1.02)	.909	1.00 (0.97–1.02)	.749				
Dose per fraction	1.50 (0.69–3.29)	.310	1.38 (0.56–3.36)	.483				
Nr. of fractions	1.00 (0.95–1.04)	.845	0.99 (0.94–1.04)	.691				
Technique								
Photons	Reference		Reference					
Protons	0.92 (0.79–1.06)	.244	0.88 (0.74–1.04)	.137				
Modality								
3D-CRT	Reference		Reference					
IMRT/VMAT	1.45 (0.54–3.88)	.462	1.89 (0.47–7.63)	.368				
Protons	1.32 (0.49–3.56)	.581	1.66 (0.41–6.72)	.477				
Modality								
IMRT	Reference		Reference					
VMAT	1.20 (1.00–1.45)	.051	1.11 (0.89–1.39)	.366				
Modality								
PSPT	Reference		Reference					
IMPT	1.24 (0.84–1.83)	.280	1.44 (0.96–2.16)	.074				
PTV volume	1.03 (1.01–1.05)	.001	1.04 (1.02–1.06)	.001				
Induction chemotherapy								
No	Reference		Reference					
Yes	1.13 (0.98–1.31)	.087	1.16 (0.99–1.36)	.061				
Surgery	0.42 (0.36–0.48)	<.001*	0.46 (0.39–0.54)	<.001*	0.35 (0.30–0.42)	<.001*	0.44 (0.37–0.53)	<.001*
					With the addition of either one of the following:			
Baseline ALC	0.91 (0.82–1.02)	.095	0.91 (0.80–1.03)	.142	0.99 (0.88–1.11)	.878	1.01 (0.89–1.15)	.878
ALCnadir	0.34 (0.22–0.52)	<.001*	0.32 (0.20–0.53)	<0.001*	0.55 (0.34–0.92)	.021	0.56 (0.34–0.92)	.021
Grade ≥3 RIL in any week	1.49 (1.16–1.92)	.002	1.49 (1.11–1.98)	.007	1.28 (0.99–1.66)	.056	1.24 (0.92–1.65)	.156
Grade 4 RIL in any week	1.26 (1.10–1.45)	.001	1.33 (1.14–1.55)	<0.001*	1.06 (0.92–1.22)	.426	1.10 (0.93–1.29)	.259
ALC wk 1	0.90 (0.74–1.09)	.288	0.92 (0.74–1.14)	0.442	0.97 (0.78–1.20)	.761	1.02 (0.80–1.30)	.841
ALC wk 2	0.64 (0.52–0.78)	<.001*	0.64 (0.51–0.80)	<0.001*	0.74 (0.60–0.91)	.004	0.77 (0.61–0.96)	.023
ALC wk 3	0.50 (0.38–0.66)	<.001*	0.50 (0.37–0.68)	<0.001*	0.60 (0.47–0.79)	<.001*	0.64 (0.48–0.87)	.004
ALC wk 4	0.49 (0.36–0.67)	<.001*	0.54 (0.38–0.77)	0.001	0.60 (0.43–0.82)	.001	0.70 (0.50–0.99)	.046
ALC wk 5	0.52 (0.36–0.75)	<.001*	0.54 (0.36–0.82)	0.003	0.73 (0.50–1.05)	.090	0.82 (0.54–1.24)	.340
ALC wk 6	0.59 (0.37–0.95)	.030	0.55 (0.33–0.92)	0.024	0.82 (0.50–1.33)	.415	0.83 (0.49–1.40)	.482
Baseline ANC	1.07 (1.04–1.10)	<.001*	1.07 (1.04–1.11)	<0.001*	1.06 (1.03–1.10)	<.001*	1.06 (1.03–1.10)	<.001*
NLR max	1.01 (1.00–1.01)	<.001*	1.01 (1.00–1.01)	<.001*	1.00 (1.00–1.01)	.049	1.00 (1.00–1.01)	.030
NLR wk 1	1.04 (1.02–1.07)	<.001*	1.04 (1.01–1.07)	.003	1.03 (1.01–1.06)	.015	1.03 (1.01–1.06)	.016
NLR wk 2	1.01 (1.01–1.02)	<.001*	1.01 (1.01–1.02)	<.001*	1.01 (1.01–1.02)	<.001*	1.01 (1.00–1.02)	.004
NLR wk 3	1.02 (1.01–1.03)	<.001*	1.02 (1.01–1.03)	<.001*	1.02 (1.01–1.02)	<.001*	1.02 (1.01–1.03)	<.001*
NLR wk 4	1.01 (1.00–1.01)	<.001*	1.01 (1.00–1.01)	.001	1.01 (1.00–1.01)	.010	1.01 (1.00–1.01)	.021
NLR wk 5	1.00 (1.00–1.01)	.012	1.00 (1.00–1.01)	.013	1.00 (0.99–1.00)	.513	1.00 (0.99–1.00)	.479
NLR wk 6	1.01 (1.00–1.01)	.028	1.01 (1.00–1.01)	.008	1.00 (0.99–1.01)	.667	1.00 (0.99–1.01)	.433
ΔALC wk 1	1.07 (0.88–1.29)	.501	1.05 (0.85–1.30)	.658	0.99 (0.79–1.24)	.947	0.99 (0.78–1.25)	.922
ΔALC wk 2	0.91 (0.79–1.05)	.182	0.93 (0.80–1.09)	.367	0.85 (0.74–0.99)	.039	0.87 (0.74–1.03)	.109
ΔALC wk 3	0.94 (0.82–1.07)	.330	0.96 (0.83–1.12)	.624	0.84 (0.73–0.96)	.011	0.88 (0.75–1.02)	.093
ΔALC wk 4	0.93 (0.82–1.05)	.243	0.96 (0.84–1.10)	.546	0.86 (0.76–0.98)	.020	0.89 (0.77–1.03)	.106
ΔALC wk 5	1.01 (0.89–1.14)	.872	1.03 (0.89–1.18)	.694	0.96 (0.84–1.09)	.510	0.98 (0.84–1.13)	.766
ΔALC wk 6	1.02 (0.87–1.19)	.817	0.97 (0.83–1.14)	.723	0.93 (0.80–1.10)	.398	0.88 (0.74–1.05)	.158
ΔALC % wk 1^†^	0.99 (0.96–1.03)	.627	0.99 (0.95–1.03)	.647	0.99 (0.95–1.02)	.451	0.99 (0.95–1.03)	.613
ΔALC % wk 2^†^	0.94 (0.91–0.97)	<.001*	0.94 (0.90–0.97)	.001	0.94 (0.91–0.98)	<.001*	0.95 (0.91–0.98)	.004
ΔALC % wk 3^†^	0.91 (0.88–0.95)	<.001*	0.91 (0.87–0.96)	<.001*	0.91 (0.87–0.95)	<.001*	0.92 (0.88–0.97)	<.001*
ΔALC % wk 4^†^	0.90 (0.86–0.94)	<.001*	0.92 (0.87–0.97)	.002	0.91 (0.86–0.95)	<.001*	0.93 (0.88–0.98)	.006
ΔALC % wk 5^†^	0.94 (0.89–0.99)	.019	0.94 (0.89–1.00)	.062	0.96 (0.91–1.01)	.114	0.97 (0.92–1.03)	.304
ΔALC % wk 6^†^	0.95 (0.90–1.01)	.128	0.93 (0.87–1.00)	.059	0.96 (0.90–1.02)	.194	0.94 (0.88–1.01)	.112

*Abbreviations:* aHR = adjusted hazard ratio; AJCC = American Joint Committee on Cancer; ALC = absolute lymphocyte count; ANC = absolute neutrophil count; BMI = body mass index; HR = hazard ratio; IMPT = intensity modulated proton therapy; IMRT = intensity modulated radiation therapy; NLR = neutrophil-to-lymphocyte ratio; Nr. = number; PSPT = passive scattering proton therapy; PTV = planning target volume; RIL = radiation-induced lymphopenia; SCC = squamous cell carcinoma; 3D-CRT = 3-dimensional conformal radiation therapy; VMAT = volumetric modulated arc therapy; WHO = World Health Organization.

* Statistically significant (*P* < .001).

† Per 10%

**Table 3:** Corrected c-statistics for a selection of the multi-variable models (ie, the best-performing models and most commonly used RIL metrics)

RIL metrics	PFS corrected c-statistic	OS corrected c-statistic
Most promising		
ALC wk 3	0.683	0.662
ΔALC (%) wk 3	0.682	0.663
Baseline ANC	0.679	0.660
NLR wk 1	0.680	0.661
NLR wk 3	0.682	0.662
Commonly applied		
Baseline ALC	0.677	0.656
Grade ≥3 RIL	0.678	0.658
Grade 4 RIL	0.677	0.657
ALCnadir	0.680	0.660

*Abbreviations:* ALC = absolute lymphocyte count; ANC = absolute neutrophil count; NLR = neutrophil-to-lymphocyte ratio; OS = overall survival; PFS = progression-free survival; RIL = radiation-induced lymphopenia.

## Data Availability

All data generated or analyzed during this study are included in this published article and its supplementary information files.

## References

[1] Sung H, Ferlay J, Siegel RL, Global cancer statistics 2020: GLO-BOCAN estimates of incidence and mortality worldwide for 36 cancers in 185 countries. CA Cancer J Clin 2021;71:209–249. 33538338 10.3322/caac.21660

[2] Eyck BM, van Lanschot JJB, Hulshof MCCM, Ten-year outcome of neoadjuvant chemoradiotherapy plus surgery for esophageal cancer: The randomized controlled CROSS trial. J Clin Oncol 2021;39:1995–2004. 33891478 10.1200/JCO.20.03614

[3] Kelly RJ, Ajani JA, Kuzdzal J, Adjuvant nivolumab in resected esophageal or gastroesophageal junction cancer. N Engl J Med 2021;384:1191–1203. 33789008 10.1056/NEJMoa2032125

[4] Hoeppner J, Lordick F, Brunner T, ESOPEC: Prospective randomized controlled multicenter phase III trial comparing perioperative chemotherapy (FLOT protocol) to neoadjuvant chemoradiation (CROSS protocol) in patients with adenocarcinoma of the esophagus (NCT02509286). BMC Cancer 2016;16:503. 27435280 10.1186/s12885-016-2564-y PMC4952147

[5] Mellman I, Coukos G, Dranoff G. Cancer immunotherapy comes of age. Nature 2011;480:480–489. 22193102 10.1038/nature10673 PMC3967235

[6] Molon B, Calì B, Viola A. T cells and cancer: How metabolism shapes immunity. Front Immunol 2016;7:20. 26870036 10.3389/fimmu.2016.00020 PMC4740780

[7] Yovino S, Grossman SA. Severity, etiology and possible consequences of treatment-related lymphopenia in patients with newly diagnosed high-grade gliomas. CNS Oncol 2012;1:149–154. 23828734 10.2217/cns.12.14 PMC3697135

[8] Sellins KS, Cohen JJ. Gene induction by gamma-irradiation leads to DNA fragmentation in lymphocytes. J Immunol 1987;139:3199–3206. 3680944

[9] Stratton JA, Byfield PE, Byfield JE, A comparison of the acute effects of radiation therapy, including or excluding the thymus, on the lymphocyte subpopulations of cancer patients. J Clin Invest 1975;56:88–97. 1095613 10.1172/JCI108084 PMC436559

[10] Dai D, Tian Q, Yu G, Severe radiation-induced lymphopenia affects the outcomes of esophageal cancer: A comprehensive systematic review and meta-analysis. Cancers (Basel) 2022;14:3024. 35740689 10.3390/cancers14123024 PMC9221375

[11] Hyder J, Boggs DH, Hanna A, Suntharalingam M, Chuong MD. Changes in neutrophil-to-lymphocyte and platelet-to-lymphocyte ratios during chemoradiation predict for survival and pathologic complete response in trimodality esophageal cancer patients. J Gastrointest Oncol 2016;7:189–195. 27034785 10.3978/j.issn.2078-6891.2015.080 PMC4783757

[12] Zhou XL, Zhu WG, Zhu ZJ, Lymphopenia in esophageal squamous cell carcinoma: Relationship to malnutrition, various disease parameters, and response to concurrent chemoradiotherapy. Oncologist 2019;24:e677–e686. 31040254 10.1634/theoncologist.2018-0723 PMC6693723

[13] Li Q, Zhou S, Liu S, Treatment-related lymphopenia predicts pathologic complete response and recurrence in esophageal squamous cell carcinoma undergoing neoadjuvant chemoradiotherapy. Ann Surg Oncol 2019;26:2882–2889. 31037433 10.1245/s10434-019-07334-7

[14] Fang P, Jiang W, Davuluri R, High lymphocyte count during neoadjuvant chemoradiotherapy is associated with improved pathologic complete response in esophageal cancer. Radiother Oncol 2018;128:584–590. 29530432 10.1016/j.radonc.2018.02.025

[15] Jing W, Xu T, Wu L, Severe radiation-induced lymphopenia attenuates the benefit of durvalumab after concurrent chemoradiotherapy for NSCLC. JTO Clin Res Rep 2022;3 100391. 36089921 10.1016/j.jtocrr.2022.100391 PMC9449658

[16] Xu H, Lin M, Hu Y, Lymphopenia during definitive chemoradiotherapy in esophageal squamous cell carcinoma: Association with dosimetric parameters and patient outcomes. Oncologist 2021;26:e425–e434. 32960471 10.1002/onco.13533 PMC7930419

[17] Deng W, Xu C, Liu A, The relationship of lymphocyte recovery and prognosis of esophageal cancer patients with severe radiation-induced lymphopenia after chemoradiation therapy. Radiother Oncol 2019;133:9–15. 30935587 10.1016/j.radonc.2018.12.002

[18] Damen PJJ, Kroese TE, van Hillegersberg R, The influence of severe radiation-induced lymphopenia on overall survival in solid tumors: A systematic review and meta-analysis. Int J Radiat Oncol Biol Phys 2021;111:936–948. 34329738 10.1016/j.ijrobp.2021.07.1695

[19] van Rossum PSN, Deng W, Routman DM, Prediction of severe lymphopenia during chemoradiation therapy for esophageal cancer: Development and validation of a pretreatment nomogram. Pract Radiat Oncol 2020;10:e16–e26. 31369887 10.1016/j.prro.2019.07.010 PMC7564893

[20] Kroese TE, Jairam J, Ruurda JP, Severe lymphopenia acquired during chemoradiotherapy for esophageal cancer: Incidence and external validation of a prediction model. Radiother Oncol 2021;163:192–198. 34453954 10.1016/j.radonc.2021.08.009

[21] Zhang E, Deng M, Egleston B, Dose to heart, spine, aorta, and body predict for severe lymphopenia and poor survival in patients undergoing chemoradiation for esophageal cancer. Int J Radiat Oncol Biol Phys 2019;105:E206–E207.

[22] Sanmamed MF, Chen L. A paradigm shift in cancer immunotherapy: From enhancement to normalization. Cell 2018;175:313–326. 30290139 10.1016/j.cell.2018.09.035 PMC6538253

[23] Tang C, Lee MS, Gomez D, Effects of chemotherapy regimen and radiation modality on hematologic toxicities in patients receiving definitive platinum-based doublet chemoradiation for non-small cell lung cancer. Am J Clin Oncol 2017;40:625–630. 26165417 10.1097/COC.0000000000000206

[24] Abravan A, Faivre-Finn C, Kennedy J, McWilliam A, van Herk M. Radiotherapy-related lymphopenia affects overall survival in patients with lung cancer. J Thorac Oncol 2020;15:1624–1635. 32553694 10.1016/j.jtho.2020.06.008

[25] Abravan A, Eide HA, Helland Å, Malinen E. Radiotherapy-related lymphopenia in patients with advanced non-small cell lung cancer receiving palliative radiotherapy. Clin Transl Radiat Oncol 2020;22:15–21. 32181373 10.1016/j.ctro.2020.02.005 PMC7063172

[26] Guo M, Li W, Li B, Prognostic value of delta inflammatory biomarker-based nomograms in patients with inoperable locally advanced NSCLC. Int Immunopharmacol 2019;72:395–401. 31030095 10.1016/j.intimp.2019.04.032

[27] Wang X, Lu J, Teng F, Yu J. Lymphopenia association with accelerated hyperfractionation and its effects on limited-stage small cell lung cancer patients’ clinical outcomes. Ann Transl Med 2019;7 385–385. 31555699 10.21037/atm.2019.07.58 PMC6736801

[28] Chen D, Verma V, Patel RR, Absolute lymphocyte count predicts abscopal responses and outcomes in patients receiving combined immunotherapy and radiation therapy: Analysis of 3 phase 1/2 trials. Int J Radiat Oncol Biol Phys 2020;108:196–203. 32036004 10.1016/j.ijrobp.2020.01.032

[29] Anon. Common Terminology Criteria for Adverse Events Version. - Google Scholar. Available at: https://ctep.cancer.gov/protocoldevelopment/electronic_applications/docs/ctcae_v5_quick_reference_5x7.pdf. Accessed 20 June 2024.

[30] Thor M, Shepherd AF, Preeshagul I, Pre-treatment immune status predicts disease control in NSCLCs treated with chemoradiation and durvalumab. Radiother Oncol 2022;167:158–164. 34942280 10.1016/j.radonc.2021.12.016 PMC9518843

[31] Li A, Mu X, He K, Prognostic value of lymphocyte-to-monocyte ratio and systemic immune-inflammation index in non-small-cell lung cancer patients with brain metastases. Future Oncol 2020;16:2433–2444. 32664750 10.2217/fon-2020-0423

[32] Park EY, Kim YS, Choi KH, Prognostic value of neutrophil-to-lymphocyte ratio in locally advanced non-small cell lung cancer treated with concurrent chemoradiotherapy. Radiat Oncol J 2019;37:166–175. 31591864 10.3857/roj.2019.00220 PMC6790800

[33] Collins GS, Reitsma JB, Altman DG, Moons KGM. Transparent reporting of a multivariable prediction model for individual prognosis or diagnosis (TRIPOD): The TRIPOD statement. Ann Intern Med 2015;162:55–63. 25560714 10.7326/M14-0697

[34] Venkatesulu BP, Mallick S, Lin SH, Krishnan S. A systematic review of the influence of radiation-induced lymphopenia on survival outcomes in solid tumors. Crit Rev Oncol Hematol 2018;123:42–51. 29482778 10.1016/j.critrevonc.2018.01.003

[35] Lambin P, Lieverse RIY, Eckert F, Lymphocyte-sparing radiotherapy: The rationale for protecting lymphocyte-rich organs when combining radiotherapy with immunotherapy. Semin Radiat Oncol 2020;30:187–193. 32381298 10.1016/j.semradonc.2019.12.003 PMC8412054

[36] Venkatesulu BP, Giridhar P, Pujari L, Lymphocyte sparing normal tissue effects in the clinic (LymphoTEC): A systematic review of dose constraint considerations to mitigate radiation-related lymphopenia in the era of immunotherapy. Radiother Oncol 2022;177:81–94. 36334694 10.1016/j.radonc.2022.10.019

[37] Lin SH, Hobbs BP, Verma V, Randomized phase IIB trial of proton beam therapy versus intensity-modulated radiation therapy for locally advanced esophageal cancer. J Clin Oncol 2020;38:1569–1579. 32160096 10.1200/JCO.19.02503 PMC7213588

[38] Davuluri R, Jiang W, Fang P, Lymphocyte nadir and esophageal cancer survival outcomes after chemoradiation therapy. Int J Radiat Oncol Biol Phys 2017;99:128–135. 28816138 10.1016/j.ijrobp.2017.05.037

[39] Wang X, Van Rossum PSN, Chu Y, Severe lymphopenia during chemoradiation therapy for esophageal cancer: Comprehensive analysis of randomized phase 2b trial of proton beam therapy versus intensity modulated radiation therapy. Int J Radiat Oncol Biol Phys 2024;118:368–377. 37652304 10.1016/j.ijrobp.2023.08.058

[40] Chen Y, Chu Y, van Rossum PSN, Radiation-induced lymphopenia is a causal mediator of survival after chemoradiation therapy for esophagus cancer. Adv Radiat Oncol 2024;9 101579. 39258141 10.1016/j.adro.2024.101579 PMC11382310

[41] Zhou P, Du Y, Zhang Y, Efficacy and safety in proton therapy and photon therapy for patients with esophageal cancer: A meta-analysis. JAMA Netw Open 2023;6 e2328136. 37581887 10.1001/jamanetworkopen.2023.28136 PMC10427943

[42] Raaymakers BW, Lagendijk JJ, Overweg J, Integrating a 1.5 T MRI scanner with a 6 MV accelerator: Proof of concept. Phys Med Biol 2009;54:N229–N237. 19451689 10.1088/0031-9155/54/12/N01

[43] Morgan HE, Wang K, Yan Y, Preliminary evaluation of PTV margins for online adaptive radiation therapy of the prostatic fossa. Pract Radiat Oncol 2023;13:e345–e353. 36509197 10.1016/j.prro.2022.11.003

[44] Favaudon V, Caplier L, Monceau V, Ultrahigh dose-rate FLASH irradiation increases the differential response between normal and tumor tissue in mice. Sci Transl Med 2014;6:245ra93. 10.1126/scitranslmed.3008973 25031268

[45] Byun HK, Kim KJ, Han SC, Seong J. Effect of interleukin-7 on radiation-induced lymphopenia and its antitumor effects in a mouse model. Int J Radiat Oncol Biol Phys 2021;109:1559–1569. 33321193 10.1016/j.ijrobp.2020.12.004

[46] Campian JL, Ye X, Gladstone DE, Pre-radiation lymphocyte harvesting and post-radiation reinfusion in patients with newly diagnosed high grade gliomas. J Neurooncol 2015;124:307–316. 26070554 10.1007/s11060-015-1841-y PMC4696006

[47] Reynolds JV, Preston SR, O’Neill B, Trimodality therapy versus perioperative chemotherapy in the management of locally advanced adenocarcinoma of the oesophagus and oesophagogastric junction (neo-AEGIS): An open-label, randomised, phase 3 trial. Lancet Gastroenterol Hepatol 2023;8:1015–1027. 37734399 10.1016/S2468-1253(23)00243-1 PMC10567579

[48] Dai D, Tian Q, Shui Y, Li J, Wei Q. The impact of radiation induced lymphopenia in the prognosis of head and neck cancer: A systematic review and meta-analysis. Radiother Oncol 2022;168:28–36. 35017020 10.1016/j.radonc.2022.01.003

[49] Upadhyay R, Venkatesulu BP, Giridhar P, Risk and impact of radiation related lymphopenia in lung cancer: A systematic review and meta-analysis. Radiother Oncol 2021;157:225–233. 33577865 10.1016/j.radonc.2021.01.034

[50] Pike LRG, Bang A, Mahal BA, The impact of radiation therapy on lymphocyte count and survival in metastatic cancer patients receiving PD-1 immune checkpoint inhibitors. Int J Radiat Oncol Biol Phys 2019;103:142–151. 30227198 10.1016/j.ijrobp.2018.09.010

[51] Friedes C, Chakrabarti T, Olson S, Association of severe lymphopenia and disease progression in unresectable locally advanced non-small cell lung cancer treated with definitive chemoradiation and immunotherapy. Lung Cancer 2021;154:36–43. 33611224 10.1016/j.lungcan.2021.01.022

[52] Yin T, Wang P, Yu J, Teng F. Treatment-related lymphopenia impairs the treatment response of anti-PD-1 therapy in esophageal squamous cell carcinoma. Int Immunopharmacol 2022;106 108623. 35203044 10.1016/j.intimp.2022.108623

[53] Zhao Q, Bi Y, Xue J, Prognostic value of absolute lymphocyte count in patients with advanced esophageal cancer treated with immunotherapy: A retrospective analysis. Ann Transl Med 2022;10 744–744. 35957729 10.21037/atm-22-2669 PMC9358517

[54] Koukourakis MI, Giatromanolaki A. Lymphopenia and intratumoral lymphocytic balance in the era of cancer immuno-radiotherapy. Crit Rev Oncol Hematol 2021;159 103226. 33482348 10.1016/j.critrevonc.2021.103226

[55] Theelen WSME, Chen D, Verma V, Pembrolizumab with or without radiotherapy for metastatic non-small-cell lung cancer: A pooled analysis of two randomised trials. Lancet Respir Med 2021;9:467–475. 33096027 10.1016/S2213-2600(20)30391-X

