# Supplementary material for: Defining the Optimal Radiation-induced Lymphopenia Metric to Discern Its Survival Impact in Esophageal Cancer
Source: Int J Radiat Oncol Biol Phys. Author manuscript; Available in PMC 2025 Jul 21. (PMC12278156; doi:10.1016/j.ijrobp.2024.12.014)
Supplement: Suppl [file NIHMS2097013-supplement-Suppl.docx]

**Supplemental Files**

**Figure E1.** Flowchart of patient selection.

Exclusion

(n = 145)

- planned total dose < 41.4Gy = 26
- concurrent immunotherapy = 6
- treated before 2004 = 1
- <90d follow-up = 19
- missing ALC values in week 4, 5 and 6 during CRT = 74
- overall CRT treatment time >50 days = 19

Included in this study
(n = 1339)

Patients eligible for study
(n = 1484)

**Search**

**Figure E2.** Kaplan-Meier analysis for PFS and OS for (A,B) baseline ALC, (C,D) ALC nadir, (E,F) ALC gr ≥3, (G,H) baseline ANC, (I,J) NLR in week 1, (K,L) NLR in week 3, (M,N) ΔALC % in week 3.


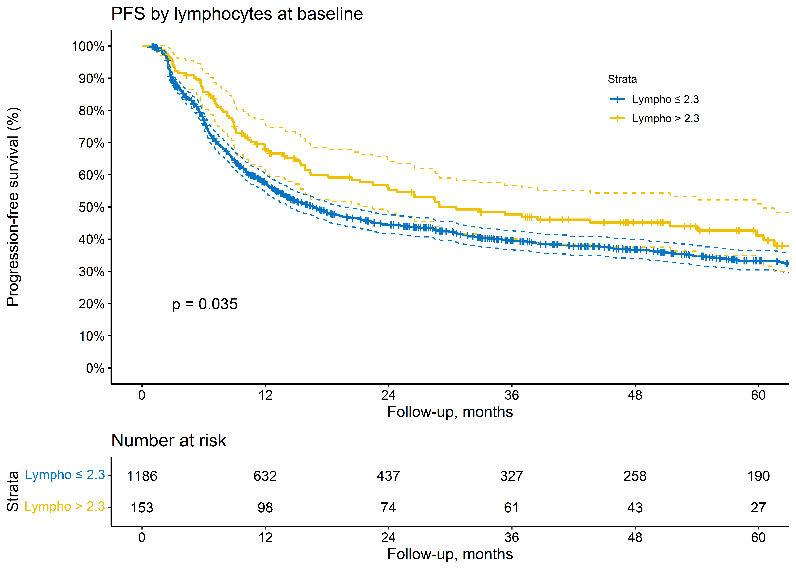

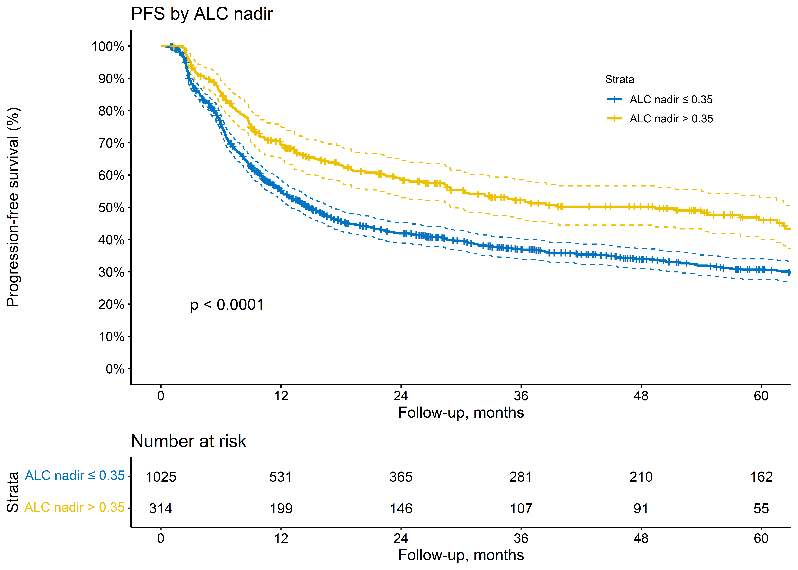

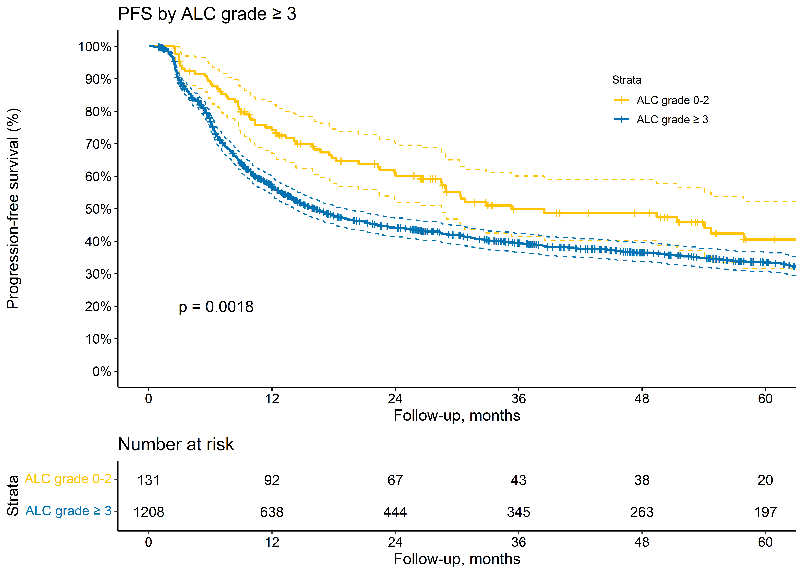

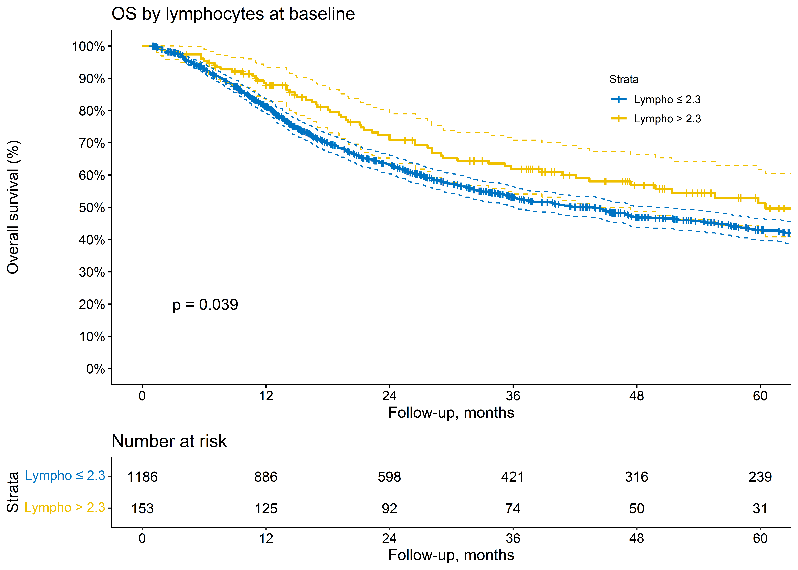

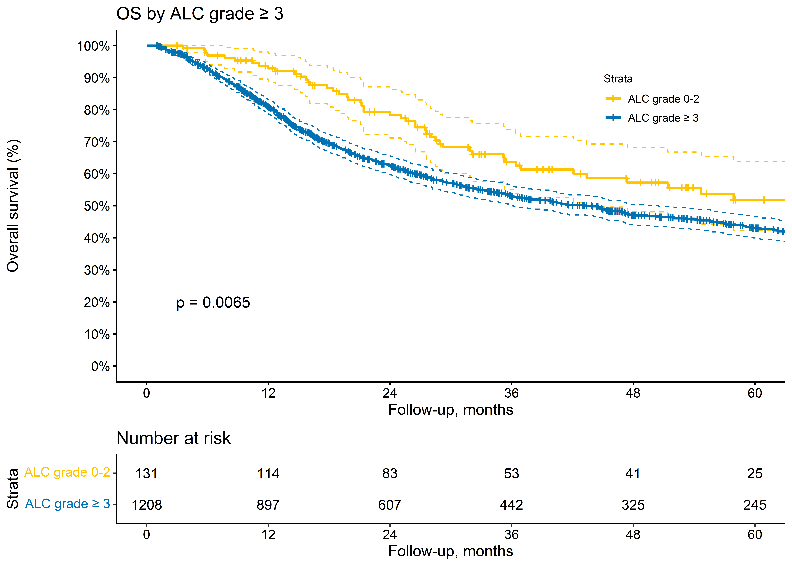

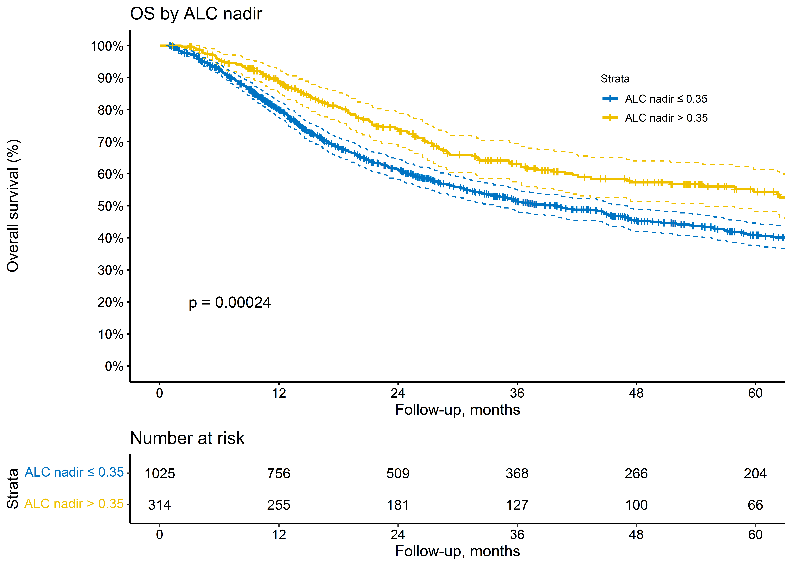


F

E

D

C

B

A


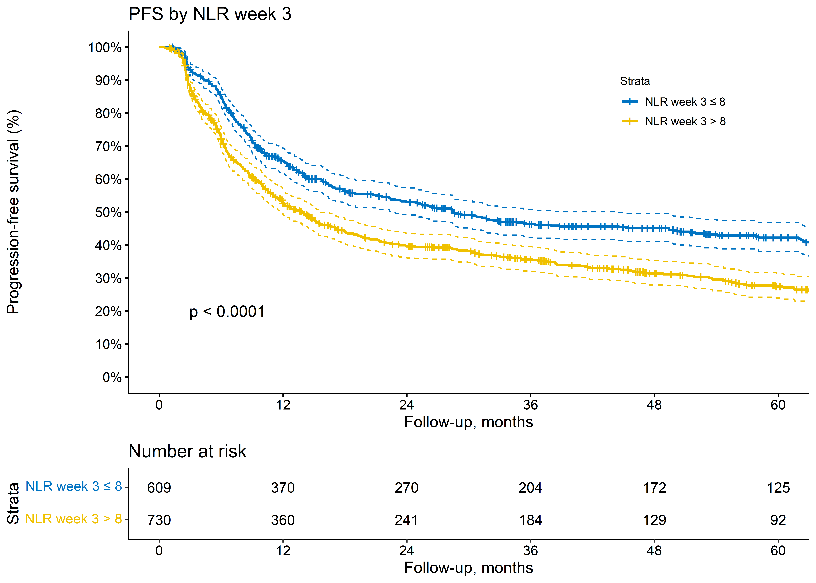

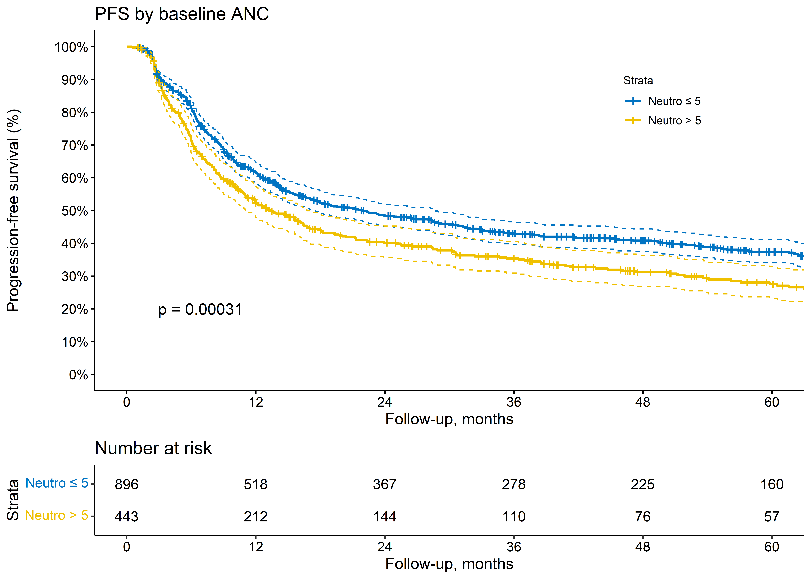

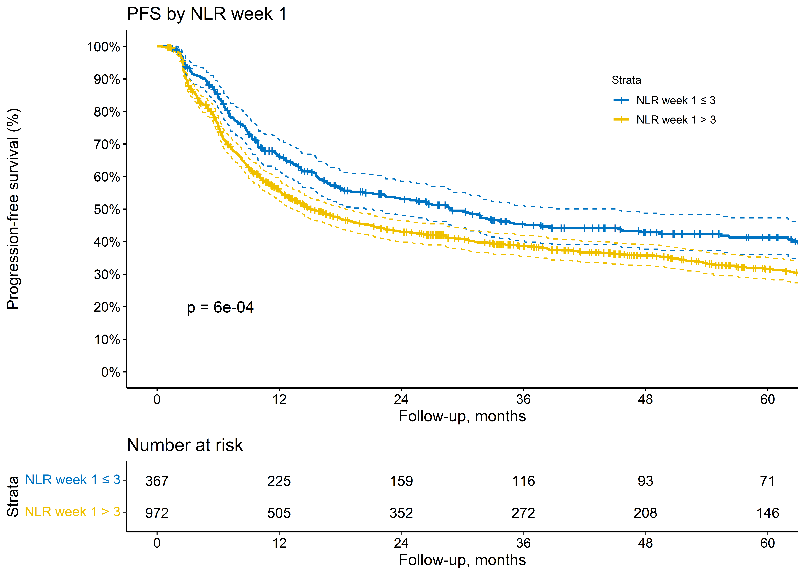

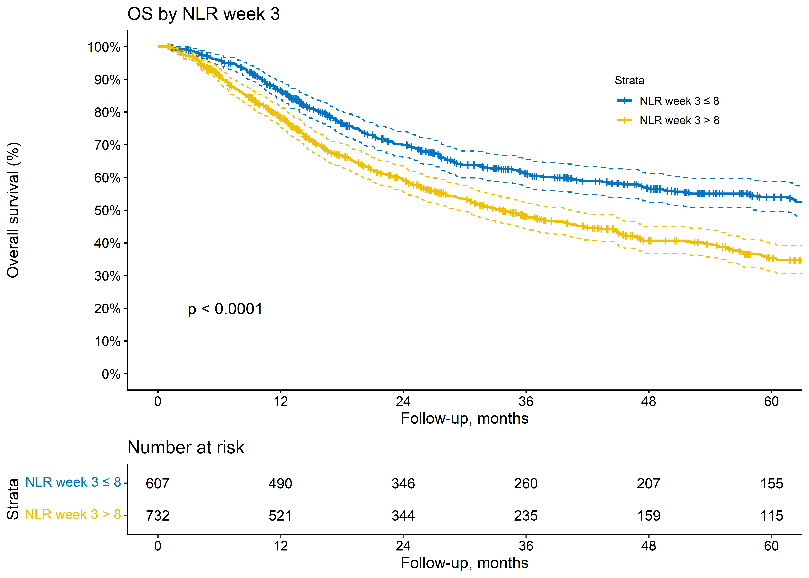

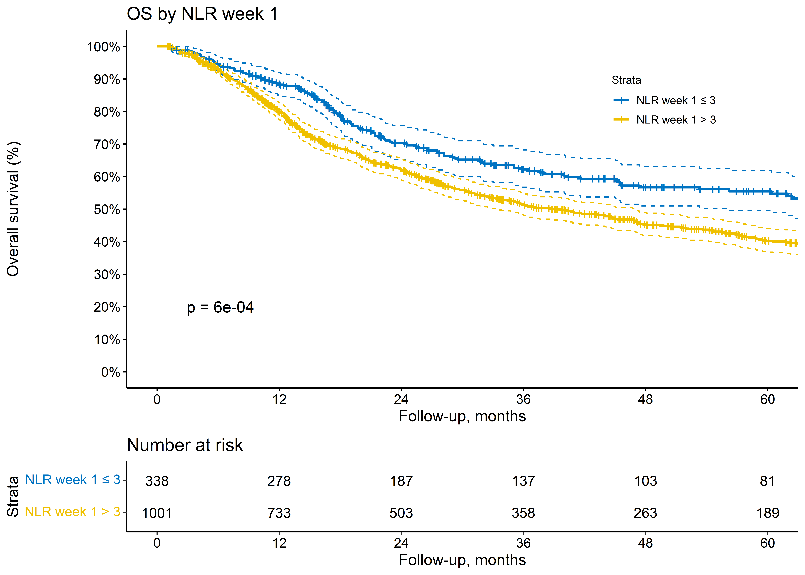

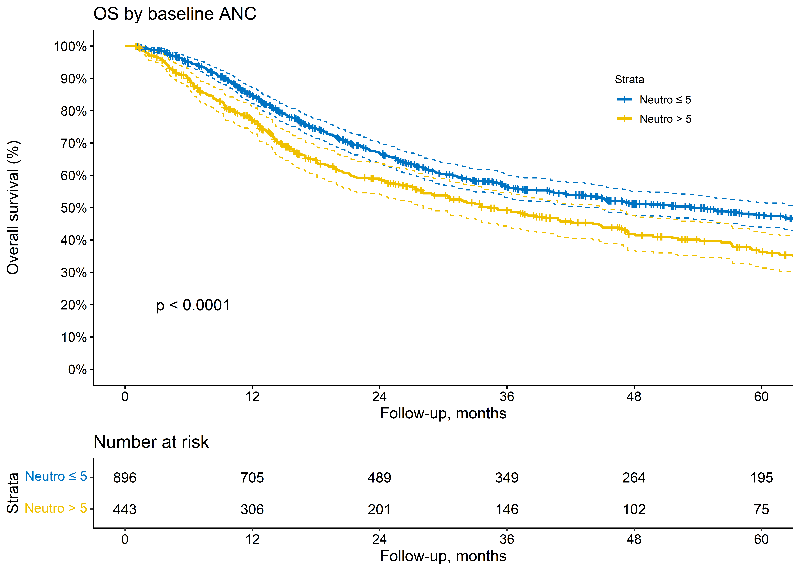


L

K

J

I

H

G


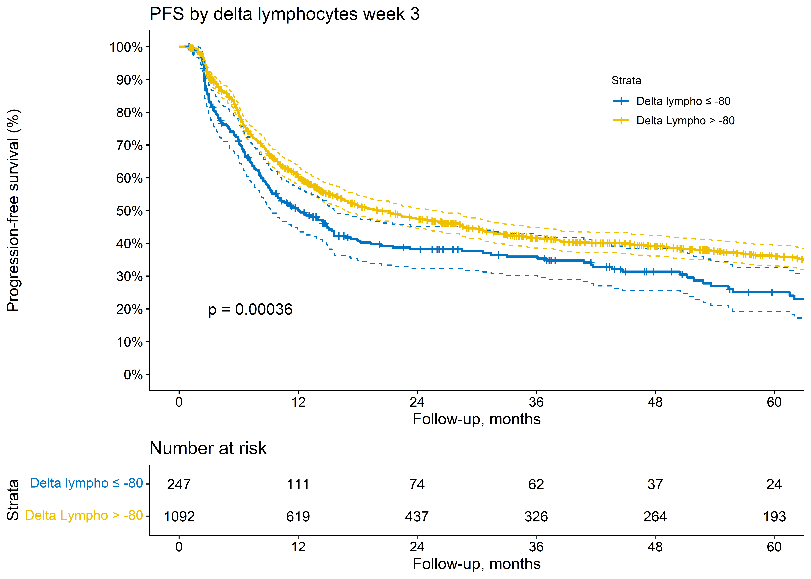

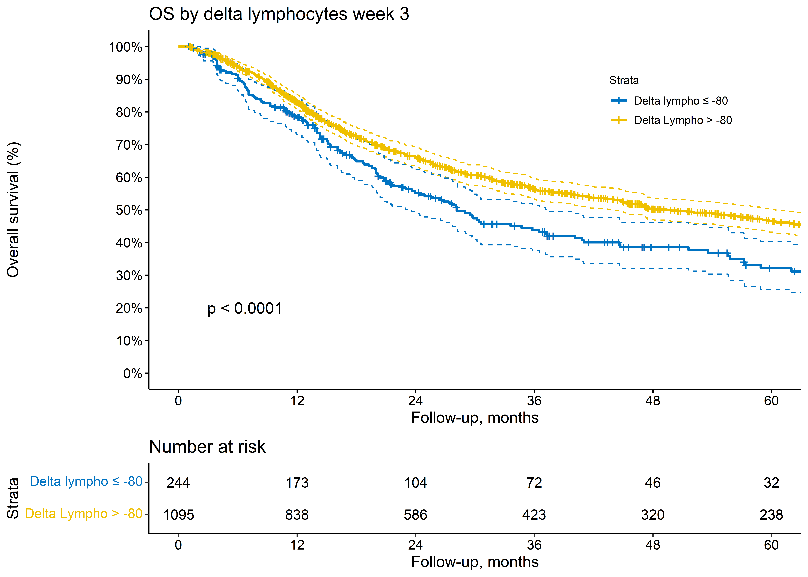


N

M

| **Table E1.** AIC values of all RIL metrics included in multivariable Cox regression models for PFS and OS. | | |
| --- | --- | --- |
|  | **PFS** | **OS** |
| **Metric** | **AIC** | **AIC** |
| Baseline ALC | 8573.86 | 10809.56 |
| ALC nadir | 8568.26 | 10799.81 |
| ALC gr ≥3 | 8571.74 | 10805.68 |
| ALC gr 4 | 8572.61 | 10808.96 |
| ALC week 1 | 8572.79 | 10808.12 |
| ALC week 2 | 8567.97 | 10799.40 |
| ALC week 3 | 8564.23 | 10793.10 |
| ALC week 4 | 8569.34 | 10797.56 |
| ALC week 5 | 8572.74 | 10806.07 |
| ALC week 6 | 8572.05 | 10807.06 |
| Baseline ANC | 8562.87 | 10797.13 |
| NLR max | 8569.72 | 10806.13 |
| NLR week 1 | 8559.51 | 10789.52 |
| NLR week 2 | 8566.95 | 10798.46 |
| NLR week 3 | 8562.58 | 10795.65 |
| NLR week 4 | 8568.65 | 10803.01 |
| NLR week 5 | 8573.23 | 10809.05 |
| NLR week 6 | 8571.71 | 10808.05 |
| ΔALC week 1 | 8572.95 | 10808.41 |
| ΔALC week 2 | 8571.13 | 10804.63 |
| ΔALC week 3 | 8570.82 | 10802.59 |
| ΔALC week 4 | 8571.06 | 10803.83 |
| ΔALC week 5 | 8573.67 | 10808.96 |
| ΔALC week 6 | 8569.61 | 10807.24 |
| ΔALC % week 1 | 8572.35 | 10807.69 |
| ΔALC % week 2 | 8564.54 | 10796.12 |
| ΔALC % week 3 | 8560.07 | 10788.09 |
| ΔALC % week 4 | 8565.43 | 10790.98 |
| ΔALC % week 5 | 8572.60 | 10806.58 |
| ΔALC % week 6 | 8567.61 | 10805.65 |
| AIC: Akaike's Information Criterion. ALC: absolute lymphocyte count. ANC: absolute neutrophil count. NLR: neutrophil-to-lymphocyte ratio. PFS: progression-free survival. OS: overall survival. | | |

| **Table E2.** Multivariable Cox proportional hazard regression analysis for PFS and OS for ALC in week 3 including patients without missing data only. | | | | | | | | |
| --- | --- | --- | --- | --- | --- | --- | --- | --- |
|  | **PFS** |  |  |  | **OS** |  |  |  |
| **Characteristic** | **aHR** | **Lower 95%-CI** | **Upper 95%-CI** | ***p*-value** | **aHR** | **Lower 95%-CI** | **Upper 95%-CI** | ***p*-value** |
| Age | 0.99 | 0.99 | 1.00 | 0.204 | 1.00 | 1.00 | 1.01 | 0.281 |
| BMI | 0.97 | 0.95 | 0.98 | **<0.001*** | 0.96 | 0.94 | 0.97 | **<0.001*** |
| Sex  Female  Male | Ref  1.64 | 1.28 | 2.09 | **<0.001*** | Ref  1.50 | 1.15 | 1.97 | 0.003 |
| Performance status  WHO 0  WHO 1  WHO 2 | Ref  0.93  1.06 | 0.78  0.78 | 1.10  1.44 | 0.406  0.697 | Ref  1.05  1.19 | 0.86  0.86 | 1.27  1.67 | 0.652  0.296 |
| Smoking status  No smoking  Past smoker  Active smoker | Ref  1.07  1.16 | 0.90  0.92 | 1.29  1.47 | 0.444  0.211 | Ref  1.11  1.18 | 0.91  0.91 | 1.36  1.53 | 0.320  0.208 |
| Histology  Adenocarcinoma  SCC  Other | Ref  0.78  4.44 | 0.62  1.83 | 0.97  10.81 | 0.029  0.001 | Ref  0.86  2.91 | 0.67  1.19 | 1.10  7.14 | 0.241  0.020 |
| Clinical T stage  T1-2  T3  T4 | Ref  1.60  2.06 | 1.23  1.28 | 2.10  3.31 | 0.001  0.003 | Ref  1.87  2.69 | 1.37  1.61 | 2.55  4.50 | **<0.001***  **<0.001*** |
| Clinical N stage  N0  N1  N2  N3 | Ref  1.32  1.52  1.42 | 1.10  1.24  0.94 | 1.60  1.88  2.15 | 0.003  **<0.001***  0.098 | Ref  1.40  1.49  1.34 | 1.14  1.19  0.82 | 1.73  1.88  2.18 | 0.002  0.001  0.244 |
| Clinical M stage  M0  M1 | Ref  1.28 | 0.93 | 1.76 | 0.126 | Ref  1.09 | 0.76 | 1.55 | 0.650 |
| Surgery | 0.34 | 0.29 | 0.41 | **<0.001*** | 0.46 | 0.38 | 0.56 | **<0.001*** |
| ALC week 3 | 0.63 | 0.29 | 0.83 | 0.001 | 0.65 | 0.48 | 0.89 | 0.007 |
| ALC: absolute lymphocyte count. BMI: body mass index. 95% CI: 95% confidence interval. WHO: World Health Organization. SCC: squamous cell carcinoma.  *: statistically significant (*p*<0.001). | | | | | | | | |

| **Table E3.**  Multivariable Cox proportional hazard regression analysis for PFS and OS for grade 4 RIL including patients without missing data only. | | | | | | | | |
| --- | --- | --- | --- | --- | --- | --- | --- | --- |
|  | **PFS** |  |  |  | **OS** |  |  |  |
| **Characteristic** | **aHR** | **Lower 95%-CI** | **Upper 95%-CI** | ***p*-value** | **aHR** | **Lower 95%-CI** | **Upper 95%-CI** | ***p*-value** |
| Age | 0.99 | 0.99 | 1.00 | 0.089 | 1.00 | 0.99 | 1.01 | 0.553 |
| BMI | 0.97 | 0.95 | 0.98 | **<0.001*** | 0.96 | 0.94 | 0.97 | **<0.001*** |
| Sex  Female  Male | Ref  1.62 | 1.28 | 2.04 | **<0.001*** | Ref  1.48 | 1.15 | 1.91 | 0.003 |
| Performance status  WHO 0  WHO 1  WHO 2 | Ref  0.94  1.02 | 0.79  0.76 | 1.11  1.38 | 0.456  0.885 | Ref  1.03  1.16 | 0.85  0.83 | 1.24  1.60 | 0.777  0.384 |
| Smoking status  No smoking  Past smoker  Active smoker | Ref  1.03  1.13 | 0.86  0.90 | 1.23  1.42 | 0.734  0.299 | Ref  1.08  1.18 | 0.88  0.92 | 1.31  1.51 | 0.469  0.396 |
| Histology  Adenocarcinoma  SCC  Other | Ref  0.77  4.31 | 0.62  1.77 | 0.95  10.48 | 0.017  0.001 | Ref  0.85  2.83 | 0.67  1.15 | 1.09  6.93 | 0.200  0.023 |
| Clinical T stage  T1-2  T3  T4 | Ref  1.69  2.29 | 1.30  1.45 | 2.19  3.62 | **<0.001***  **<0.001*** | Ref  1.91  2.84 | 1.42  1.73 | 2.60  4.66 | **<0.001***  **<0.001*** |
| Clinical N stage  N0  N1  N2  N3 | Ref  1.31  1.46  1.45 | 1.09  1.19  0.97 | 1.57  1.80  2.17 | 0.004  **<0.001***  0.069 | Ref  1.38  1.46  1.4 | 1.13  1.17  0.88 | 1.70  1.83  2.24 | 0.002  0.001  0.160 |
| Clinical M stage  M0  M1 | Ref  1.31 | 0.96 | 1.79 | 0.090 | Ref  1.06 | 0.74 | 1.51 | 0.751 |
| Surgery | 0.34 | 0.29 | 0.41 | **<0.001*** | 0.45 | 0.38 | 0.55 | **<0.001*** |
| Grade 4 RIL in any week | 1.09 | 0.93 | 1.27 | 0.289 | 1.14 | 0.97 | 1.35 | 0.117 |
| ALC: absolute lymphocyte count. BMI: body mass index. 95% CI: 95% confidence interval. WHO: World Health Organization. RIL: radiation-induced lymphopenia. SCC: squamous cell carcinoma.  *: statistically significant (*p*<0.001). | | | | | | | | |
